# Antibiotic hybrids: A promising strategy to replenish the pipeline and combat antimicrobial resistance

**DOI:** 10.71150/jm.2510006

**Published:** 2026-02-25

**Authors:** Yeongseo Lee, Yeo Jin Kim, Minhee Oh, Joon-Ho Lee, Saemee Song, Jaesung Kwak

**Affiliations:** 1 Infectious Diseases Therapeutic Research Center, Korea Research Institute of Chemical Technology (KRICT), Daejeon 34114, Republic of Korea; 2 Division of Medicinal Chemistry and Pharmacology, KRICT School, University of Science and Technology (UST), Daejeon 34114, Republic of Korea; 3 School of Pharmacy, Sungkyunkwan University, Suwon 16419, Republic of Korea; 4 Graduate School of New Drug Discovery and Development, Chungnam National University, Daejeon 34134, Republic of Korea

**Keywords:** antimicrobial resistance, antibiotic development, antibiotic hybrids

## Abstract

Antimicrobial resistance (AMR) poses an ongoing threat to global health, with the number of deaths directly attributable to AMR projected to rise to 8 million. One of the main reasons for the current crisis is the depletion of antibiotic candidates in clinical pipelines. To address this, more preclinical candidates must be advanced into development. However, the scientific challenges and limited economic incentives associated with antibiotic research have further aggravated the situation. Antibiotic hybrids, which combine two antibiotics with different modes of action, have emerged as a promising strategy to overcome AMR and are already being developed for clinical use. This approach takes advantage of the strong selective pressure exerted when two bactericidal agents act simultaneously. Importantly, because hybrids are administered as a single chemical entity, they may offer advantages over conventional combination therapies, such as simplified pharmacokinetics and dosing. Furthermore, since clinically validated antibiotics are used as the building blocks of hybrids, this strategy provides an efficient platform for generating new lead compounds. Recently, the concept of antibiotic hybrids has expanded beyond antibiotic–antibiotic conjugates to include the attachment of functional molecules designed to mitigate the disadvantages of the parent antibiotics. In this review, we summarize the definition of antibiotic hybrids, highlight representative compounds that have entered clinical evaluation, and discuss recent advances in their development.

## Introduction

The discovery of antibiotics has greatly improved our life expectancy by enabling the treatment of bacterial infections and providing the foundation for modern medical practices. However, the use of antibiotics inevitably leads to antimicrobial resistance (AMR), which neutralizes the effectiveness of current treatments. A 2022 systematic analysis of global mortality in 2019 revealed that 1.27 and 4.95 million deaths were attributable to and associated with AMR, respectively (Murray et al., 2022). Forecasts suggest that AMR-associated deaths will increase to 8 million by 2050, highlighting the severity of this crisis (Naghavi et al., 2024). Significant efforts have been made to reduce unnecessary antibiotic use; however, global crises such as COVID-19 and ongoing military conflicts accelerate AMR accumulation. For example, the antibiotic usage for patients hospitalized with COVID-19 surged owing to prophylactic treatment. This led to a 20% increase in resistance among six major pathogens in the U.S. between 2019 and 2022 (Centers for Disease Control and Prevention, 2024). Similarly, a hypervirulent pan-drug-resistant *Klebsiella pneumoniae* was isolated from casualties of the Ukraine war (Ljungquist et al., 2024). These findings indicate that future global catastrophic events could worsen the AMR threat.

Unlike viral pandemics like COVID-19, AMR spread is a gradual, usually invisible process, sometimes referred to as a “slow burner” (Hardie, 2020). This slow progression causes the AMR threat to be underestimated, leading drug developers and policymakers to underestimate its importance, overshadowed by more immediate crises. Effectively combating AMR requires the timely development of new therapeutic options before currently available antibiotics lose their utility. This can be achieved either by optimizing and repurposing existing agents or by discovering antibiotics that act through entirely new mechanisms. In addition, alternative biological strategies such as bacteriophage therapy, antimicrobial peptides, and immunotherapy should be considered as complementary approaches. However, despite these urgent needs, the number of antibiotics in the clinical development pipeline remains far too limited to counter the accelerating global threat posed by AMR (Prasad et al., 2022). This shortage stems from the fact that developing antibiotics offers limited financial incentives to pharmaceutical companies (Plackett, 2020). Antibiotics are prescribed for short durations and reserved as last-line treatments, making them less profitable. Consequently, many major pharmaceutical companies have discontinued their antibiotic development programs. Given that developing a new antibiotic takes an average of 14 years, keeping pace with the evolving threat of bacterial infections is nearly impossible. Thus, innovative and sustainable methods for discovering effective antimicrobial compounds are crucial in combating the growing threat of AMR. In this review, we highlight the importance of antibiotic hybrids, a strategy that combines two antimicrobial agents into a single compound. We summarize their clinical development from early stages to the present and discuss recent innovations published since 2021.

## Challenges and Strategies of Antibacterial Drug Discovery

### Small-molecule-based strategies to combat antibiotic resistance

Developing new drugs is the most immediate solution to antibiotic resistance; however, it is extremely challenging. The pace of antibiotic development has not kept up with the emergence of resistant bacterial strains, leaving clinicians with fewer effective options. A significant gap in the discovery of new antibiotics has persisted since the early 1980s. Most antibiotics introduced in the past three decades are derivatives of previously known drugs (Bergkessel et al., 2023; Theuretzbacher et al., 2020). Current research in antibacterial therapies is shifting toward alternative approaches, such as vaccines, phage therapy, and biologics, including antimicrobial peptides (Thomas and Wessel, 2022). As of 2022, 17 of 64 antibacterial therapies in clinical development are biologics. Despite this shift, small-molecule antibiotics remain one of the most successful medical innovations in history. With over 30 small-molecule antibiotics on the World Health Organization (WHO)’s list of essential medicines, their high efficacy, ease of use, and affordability make them indispensable to modern medicine. Evaluating infectious disease patterns and treatment advancements reveals that small-molecule drugs are the most practical option for addressing the majority of medical needs (Bergkessel et al., 2023).

The small-molecule-based strategy addresses antibiotic resistance through the modification of existing drugs, combination therapies with antibiotic adjuvants, high-throughput library screenings, including in silico approaches, to identify new drug candidates, exploration of natural sources, and the integration of advanced delivery systems and target-specific technologies to enhance existing treatments. In this section, we provide a brief overview of the combination method, one of the most prominent strategies for enhancing the efficiency of existing small-molecule antibiotics.

## Combination Therapy

### Definition of combination methods

The rapid escalation of antibiotic resistance, together with the high financial burden of antibiotic R&D, the low return on investment for new agents, and the growing healthcare and societal costs attributable to antimicrobial resistance, highlights the urgent need for more sustainable therapeutic strategies. Over the past three decades, the discovery of new antibiotics has slowed significantly, owing to reduced investment from major pharmaceutical companies, depletion of readily accessible compounds, and continued reliance on existing drug libraries (McKenna, 2020). The lack of innovative development strategies and over-reliance on currently available antibiotics further exacerbate this issue. Advancing new antibiotic development remains critical; however, it is equally important to maximize the therapeutic potential of existing agents. One promising approach is the use of combination therapies, which can help counteract multidrug resistance and restore the efficacy of existing antibiotics. These therapies can involve either antibiotic-antibiotic combinations or antibiotic-non-antibiotic combinations.

### Combination of two or more antibiotics

Antibacterial combination therapies can enhance effectiveness and suppress resistance through independent or synergistic mechanisms, depending on how the agents interact. Notably, combination therapies have become crucial for managing multidrug-resistant (MDR) Gram-negative bacterial infections (Tamma et al., 2012; Worthington and Melander, 2013). For example, a combination of colistin with other antibiotics is superior for treating *Pseudomonas aeruginosa* and *Acinetobacter baumannii* infections (Petrosillo et al., 2008).

Antibiotic combination therapy can be classified into three categories (Worthington and Melander, 2013): (i) targeting distinct pathways, as demonstrated by the combination of isoniazid, rifampicin, ethambutol, and pyrazinamide for treating *Mycobacterium tuberculosis*; (ii) targeting sequential steps within the same pathway, exemplified by the use of sulfamethoxazole and trimethoprim (co-trimoxazole) to inhibit consecutive steps in folic acid biosynthesis; and (iii) targeting the same molecule through different mechanisms, as seen with streptogramins.

Specifically, in *Helicobacter pylori* treatment, where monotherapy fails to achieve complete eradication, combination therapy prevents the development of resistance during treatment (De Francesco et al., 2017; Siedentop et al., 2024). A regimen of metronidazole, omeprazole, and clarithromycin successfully eradicated *H. pylori* in 88% of cases, including 90% of patients who had previously experienced treatment failure (Yousfi et al., 1995). Current guidelines recommend a concomitant therapy consisting of a proton-pump inhibitor (PPI), clarithromycin, amoxicillin, and a nitroimidazole (tinidazole or metronidazole) for 10–14 days to effectively treat *H. pylori* infections (Yaxley and Chakravarty, 2014). Similarly, combination therapy is the standard of care for other bacterial pathogens, including *M. tuberculosis* (Singh et al., 2019) and *Mycobacterium leprae* (Belachew and Naafs, 2019). Multidrug therapy is essential for tuberculosis because single agents cannot sterilize *M. tuberculosis* populations that persist across diverse lesion compartments and metabolic states (Lenaerts et al., 2015; Wallis et al., 2016). During infection, bacilli occupy heterogeneous pulmonary lesions, intracellular niches, and physiologically distinct microenvironments, leading to substantial variability in drug penetration, target engagement, and antimicrobial susceptibility (Dartois, 2014; Dartois and Barry, 2013). These complexities necessitate the use of antibiotic combinations to ensure that effective drug exposure is achieved across all infected sites. For drug-sensitive tuberculosis, the standard regimen consists of a four-drug combination (isoniazid, rifampicin, pyrazinamide, and ethambutol) administered for two months, followed by isoniazid and rifampicin for an additional four months (Kerantzas and Jacobs, 2017; Tiberi et al., 2018; World Health Organization, 2020). For drug-resistant tuberculosis, systematic and rational exploration of optimized multidrug regimens remains essential to identify more effective therapeutic strategies (Larkins-Ford and Aldridge, 2022). However, the effect of antibiotic combination therapy on resistance remains inconsistent across different pathogens, and the debate continues as to whether combination therapy may be more harmful than monotherapy. Potential harm could arise from antagonism, where antimicrobials interfere with each other's actions or metabolism (Lepper and Dowling, 1951). In addition to these concerns, combination therapy carries several other drawbacks, including higher costs, increased dosing complexity, greater risk of adverse drug reactions, and disruption of colonization resistance (Woods and Read, 2023). In a recent study, random-effects models and meta-regression analyses were applied to data from 42 eligible trials. Findings revealed no strong evidence to suggest that combination therapies either confer a benefit or pose a risk to patients in terms of resistance development, despite their widespread use in clinical practice, especially for pathogens with a high resistance potential, such as *H. pylori* and *M. tuberculosis* (Siedentop et al., 2024).

**Combination of non-antibiotic adjuvants:** Adjuvants are substances co-administered with antibiotics to boost their antimicrobial effectiveness, by either enhancing their activity or targeting antibiotic resistance mechanisms (Gill et al., 2015; Silver, 2011; Ventola, 2015). This is achieved by reducing the minimum inhibitory concentration (MIC) needed to kill bacteria, aiding in the preservation of existing antibiotic treatments (Laws et al., 2019; Melander and Melander, 2017). The synergistic effect of combining antibiotics with adjuvants can result in a therapeutic outcome that surpasses the effect of each agent used individually (Gill et al., 2015).

Only a few antibiotic–adjuvant combinations have achieved clinical success, with Augmentin, amoxicillin plus clavulanic acid, representing the first clinically successful pairing (Finlay et al., 2003). Beyond β-lactamase inhibition, clavulanate can enhance amoxicillin activity by promoting polymorphonuclear neutrophils (PMN) uptake and intracellular killing (Cuffini et al., 1996; Gómez-Lus et al., 1997; Martín et al., 1997; Pascual et al., 1989), effects relevant in infections where PMNs are central to host defense (Kadioglu et al., 2000).

Subsequent advances have led to multiple β-lactam/β-lactamase inhibitor (BL/BLI) combinations that address the growing challenge of MDR Gram-negative pathogens (Alarcia-Lacalle et al., 2025). Among these, avibactam approved with ceftazidime and aztreonam has demonstrated broad clinical and microbiologic efficacy across complicated urinary tract infections (cUTI), complicated intra-abdominal infections (cIAI), acute pyelonephritis (AP), hospital-acquired pneumonia (HAP), and ventilator-associated pneumonia (VAP) (Boattini et al., 2023; Cornely et al., 2020; Goncette et al., 2021; Nichols et al., 2018; Soriano et al., 2023). It has also shown value as salvage therapy for MDR Gram-negative bone and joint infections, although reduced activity is reported against chromosomal AmpC producers. Notably, aztreonam/avibactam expands coverage to MBL-producing organisms (Carmeli et al., 2025; Nichols et al., 2018).

Among emerging BL/BLI options, relebactam with imipenem/cilastatin restores imipenem susceptibility in resistant Enterobacterales, improving outcomes across complicated infections (Fratoni et al., 2022; Lucasti et al., 2016). Meropenem/vaborbactam shows strong efficacy in carbapenem-resistant *Enterobacteriaceae* bacteremia, cUTI/AP, HAP/VAP, and cIAI, surpassing best available therapy in cure and mortality outcomes with enhanced pathogen eradication in cUTI (Ackley et al., 2020; Kaye et al., 2018; Wunderink et al., 2018). Cefepime/enmetazobactam demonstrated superiority to piperacillin/tazobactam in Phase 3 cUTI and pyelonephritis studies (Kaye et al., 2022). Sulbactam/durlobactam, a next-generation option for carbapenem-resistant *Acinetobacter*, was non-inferior to colistin and showed markedly better tolerability (Kaye et al., 2023). Sulfone inhibitors such as tazobactam remain clinically relevant mainly with piperacillin and ceftolozane. By contrast, evidence for cefepime/tazobactam is limited to a single study in UTIs, with no demonstrated benefit for cefotaxime/tazobactam (Kaur et al., 2014). Zidebactam stands out as a promising next-generation DBO through its combined β-lactamase inhibition and high-affinity PBP2 binding, with strong early activity against MDR Enterobacterales and *P. aeruginosa* (Moya et al., 2017).

Together, these developments illustrate how modern BL/BLI combinations have substantially broadened efficacy against extended-spectrum β-lactamase (ESBL)-, AmpC β-lactamase-, *Klebsiella pneumoniae* carbapenemase (KPC)-, and oxacillinase-48 (OXA-48)-producing organisms and MDR *Pseudomonas* and *Acinetobacter*, reinforcing their critical role in combating β-lactam resistance.

Despite significant advances in antimicrobial development, current agents remain vulnerable to resistance mechanisms driven by efflux pump overexpression. Because efflux-mediated resistance reduces intracellular antibiotic concentrations regardless of chemical scaffold or mode of action, there is growing interest in efflux pump inhibitors (EPIs) as complementary adjuvants capable of restoring antibiotic activity across multiple drug classes. In contrast to BL/BLI combinations, which restore activity by protecting β-lactam scaffolds from enzymatic degradation, EPIs enhance the intracellular bioavailability of structurally diverse antibiotics and therefore represent a mechanistically distinct strategy with broad therapeutic potential.

Several EPIs have demonstrated substantial experimental activity in vitro, although none have yet reached clinical application. Among synthetic EPIs, the peptidomimetic PAβN markedly decreases the MICs of fluoroquinolones and macrolides in *P. aeruginosa* overexpressing MexAB-OprM, with reductions of up to 64-fold reported in efflux-overproducing strains (Lomovskaya et al., 2001). Similarly, the protonophore carbonyl cyanide m-chlorophenylhydrazone (CCCP) collapses the proton-motive force (PMF) and restores susceptibility to β-lactams and fluoroquinolones in *A. baumannii*, although toxicity greatly limits its therapeutic potential (Osei Sekyere and Amoako, 2017). Additional synthetic EPIs such as 1-(1-naphthylmethyl)piperazine (NMP), MBX2319 inhibit efflux through diverse mechanisms including pump assembly disruption, deep-pocket AcrB binding, MexB-selective noncompetitive inhibition, and on-target suppression of AcrAB-TolC activity leading to significant reductions in MICs for multiple antibiotic classes (Schumacher et al., 2006; Vargiu et al., 2014). A number of natural-product EPIs also exhibit promising activity. Piperine decreases ciprofloxacin MICs up to 4-fold in *S. aureus* by inhibiting NorA-mediated efflux (Khan et al., 2006), while flavonoids such as silybin and curcumin increase intracellular antibiotic accumulation by downregulating efflux-associated gene expression (Chan et al., 2011; Wang et al., 2018). Luteolin disrupts ATP synthesis and proton-gradient homeostasis to inhibit macrolide efflux in *Trueperella pyogenes* (Guo et al., 2022), whereas baicalin interferes with PMF-driven pumps in multiple Gram-positive organisms (Wang et al., 2019). Boeravinone B further acts as a direct inhibitor of the NorA pump in *S. aureus*, reducing efflux activity and enhancing antibiotic susceptibility (Singh et al., 2017).

Taken together, these experimental EPIs broaden the conceptual landscape of antimicrobial adjuvant development. By enhancing intracellular drug exposure through inhibition of active efflux, EPIs complement existing therapeutic strategies and offer a mechanistically orthogonal approach to overcoming multidrug resistance. Although toxicity, stability, and pharmacokinetic limitations currently hinder clinical translation, the diverse mechanisms observed among synthetic and natural EPIs underscore their considerable potential as future antibiotic adjuvants and reinforce the need for continued efforts toward clinically viable efflux inhibition strategies.

## Antibiotic Hybrid Strategy

### Definition of antibiotic hybrids

**Fig. 1.**
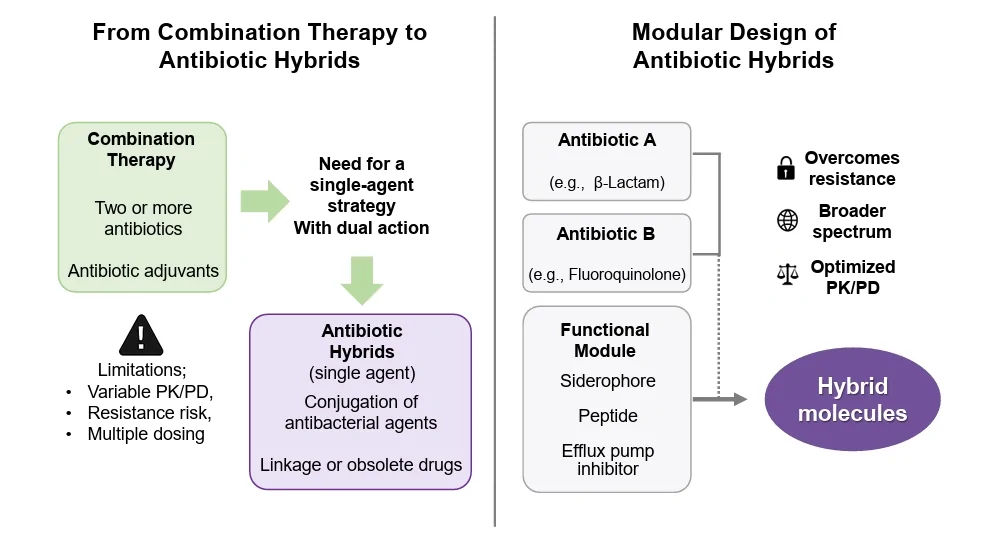
Rationale and Design Principles of Antibiotic Hybrids.

Antibiotic hybrids are a novel class of therapeutics generally defined as conjugation products of two distinct antimicrobial agents (Fig. 1). This approach has emerged as a promising solution to the challenges posed by traditional combination therapies (Domalaon et al., 2018). The term ‘dual-action antibiotics’ was initially used to describe this class of antibiotics; however, it has been replaced by ‘antibiotic hybrids.’ Upon administration, antibiotic hybrids are considered a single agent, offering several advantages over combination therapy in assessing pharmacokinetics (PK), pharmacodynamics (PD), and dose optimization for clinical trials. Recently, the scope of antibiotic hybrids has been extended to include the integration of functional modules with antibiotics. Functional modules are defined as any molecules that exert biological actions on bacteria, such as siderophores, which are used for iron uptake in prokaryotes, and cationic peptides, which disrupt bacterial membranes. The bimodality of antibiotic hybrid compounds restores the effectiveness of antibiotics that have become obsolete owing to high resistance. Additionally, antibiotics previously limited to Gram-positive bacteria due to poor permeability can be redesigned for use against Gram-negative bacteria with the proper design of hybrid compounds. The antibiotic hybrid strategy is based on clinically proven antimicrobial compounds, enabling rapid development of new antibiotics through the combination of drugs or functional molecules.

## Recent Development of Antibiotic Hybrids (2021–2025)

### β-Lactam-based antibiotic hybrids

**Fig. 2.**
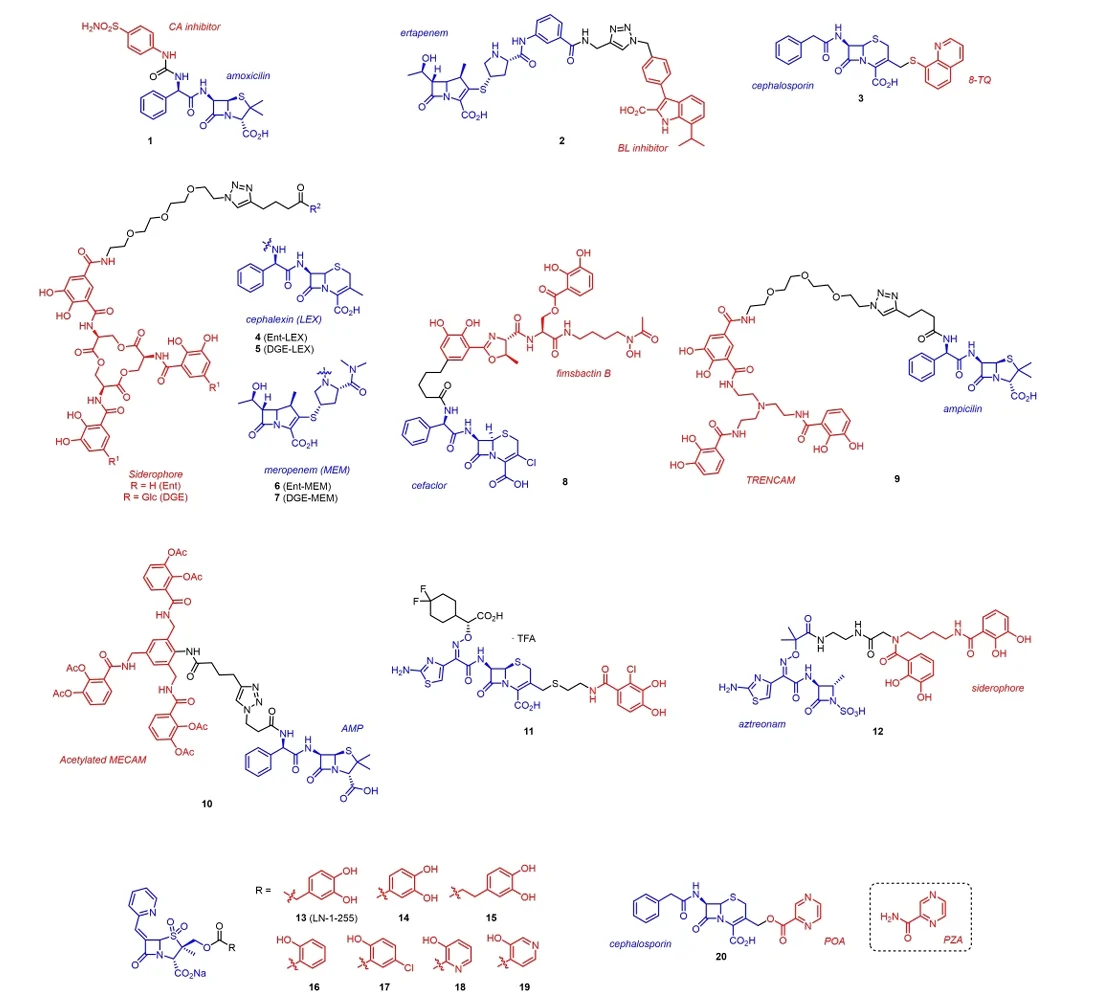
Recent examples of β-lactam-based antibiotic hybrids (2021–2025).

The β-lactam ring remains the most privileged scaffold in hybrid antibiotic design due to its validated safety profile and periplasmic target accessibility. Recent developments in this class have primarily focused on three distinct strategies to overcome antimicrobial resistance: (i) incorporating enzyme inhibitors to counteract degradation mechanisms or alternative metabolic pathways, (ii) utilizing siderophore-mediated active transport to breach the outer membrane (OM) barrier of Gram-negative pathogens, and (iii) employing prodrug mechanisms for targeted intracellular release in mycobacteria. Representative compounds and their comparative efficacy data are summarized in Table 1and Fig. 2.

A major approach involves conjugating β-lactams with enzyme inhibitors to restore susceptibility against resistant strains. Bonardi et al. (2024) introduced Compound 1, a hybrid of amoxicillin and a carbonic anhydrase inhibitor (CAI) linked via a urea bond. While the β-lactam moiety targets penicillin-binding proteins (PBPs), the CAI component disrupts the bicarbonate transport essential for bacterial growth. This dual-targeting approach allowed Compound 1 to maintain potent antimicrobial activity against multidrug-resistant *Neisseria gonorrhoeae*, demonstrating the synergistic potential of combining distinct pharmacophores. In the context of carbapenem resistance, Gao et al. (2024) synthesized carbapenem-metallo-β-lactamase (MBL) inhibitor conjugates, exemplified by Compound 2. Although these hybrids exhibited superior enzymatic inhibition against NDM-1 and IMP-1 MBLs compared to the inhibitor alone, they failed to show significant cell-based antibacterial activity against MBL-producing Gram-negative bacteria. This discrepancy was attributed to reduced cellular uptake or periplasmic accumulation, highlighting the critical importance of linker optimization in hybrid design. Additionally, addressing the challenge of MBL toxicity, van Haren et al. (2021) designed a prodrug, Compound 3, by conjugating the zinc chelator 8-thioquinoline (8-TQ) to the C3' position of a cephalosporin. This hybrid releases 8-TQ locally at the infection site upon hydrolysis by MBLs, effectively inhibiting the enzyme while mitigating systemic toxicity, thereby restoring the efficacy of co-administered meropenem against MBL-producing *K. pneumoniae* and *E. coli*.

In parallel with enzyme inhibition strategies, siderophore conjugation continues to be a dominant "Trojan horse" strategy for facilitating the active transport of β-lactams across the OM of Gram-negative bacteria. Sargun et al. (2021) investigated the selectivity of siderophore receptors using enterobactin (Ent) and diglucosylated enterobactin (DGE) conjugates (Compounds 4–7). Their study revealed that the meropenem-enterobactin conjugate (Compound 6) utilized both FepA and IroN receptors for efficient uptake, whereas DGE-based analogues relied selectively on the IroN receptor. Consequently, DGE-conjugates selectively targeted pathogenic *E. coli* strains harboring the *iroA* gene cluster while sparing commensal strains lacking this receptor. Similarly, utilizing a native siderophore strategy, Kim and Kim (2021) conjugated cefaclor with fimsbactin B, a siderophore specific to *A. baumannii*, yielding Compound 8. This hybrid demonstrated potent activity (MIC = 0.5 μg/ml) under iron-deficient conditions against receptor-positive strains, whereas cefaclor alone was ineffective (MIC = 64 μg/ml). However, its lack of activity against strains missing the fimsbactin gene cluster underscores the high species-specificity of native siderophore-based strategies. Synthetic siderophore mimics have also gained attention for their stability and synthetic feasibility. Motz et al. (2024) utilized the TRENCAM scaffold to create Compound 9, which mimicked the uptake pathway of natural enterobactin via FepA and IroN receptors, achieving uptake efficiency comparable to the natural siderophore. Similarly, Pinkert et al. (2021) employed a MECAM-based scaffold to develop Compound 10. Notably, transcriptomic analysis indicated that high concentrations of Compound 10 induced an SOS response and prophage expression, leading to a paradoxical bacterial regrowth known as the "Eagle effect" (Lai et al., 2023).

Optimization of clinical candidates and linker technologies is also actively being pursued within siderophore strategies. Liu et al. (2024) developed Compound 11, a catechol-based siderophore-cephalosporin hybrid designed to improve upon cefiderocol. Through modifications to the linker and aminothiazole moiety, Compound 11 demonstrated enhanced potency against MDR *A. baumannii* and *K. pneumoniae* compared to cefiderocol, while maintaining a comparable pharmacokinetic profile. In a streamlined approach, Liu et al. (2021) synthesized Compound 12 by directly incorporating a bis-catechol siderophore into the aztreonam side chain. Despite the inherent instability of the monobactam ring, the rapid siderophore-mediated uptake of this conjugate resulted in potent inhibition of *A. baumannii* and *P. aeruginosa*. Rodríguez et al. (2024) further conducted a detailed structure–activity relationship (SAR) investigation on penicillin-based sulfone–siderophore conjugates (Compounds 13–19). They demonstrated that the catechol-bearing analogue, Compound 14, displays superior restoration of β-lactam antibiotic activity compared to phenol- or pyridyl-based counterparts. This enhancement arises from more efficient periplasmic delivery and the formation of additional stabilizing interactions within β-lactamase active sites, rather than from intrinsic antibacterial effects (Rodríguez et al., 2024).

Finally, the hybrid strategy has been extended to address specific challenges in tuberculosis treatment. Cole et al. (2022) developed Compound 20, a cephalosporin-pyrazinoic acid (POA) conjugate. Pyrazinamide (PZA) typically requires activation by the bacterial enzyme PncA to form the active POA, and mutations in *pncA* are a primary driver of resistance. Compound 20 is designed to release POA directly upon β-lactamase-mediated cleavage within the mycobacterial cell, effectively bypassing the requirement for PncA activation. This strategy successfully retained activity against PZA-resistant *M. tuberculosis* strains, offering a novel approach to overcoming resistance in mycobacteria.

### Non-β-lactam-based antibiotic hybrids

**Fig. 3.**
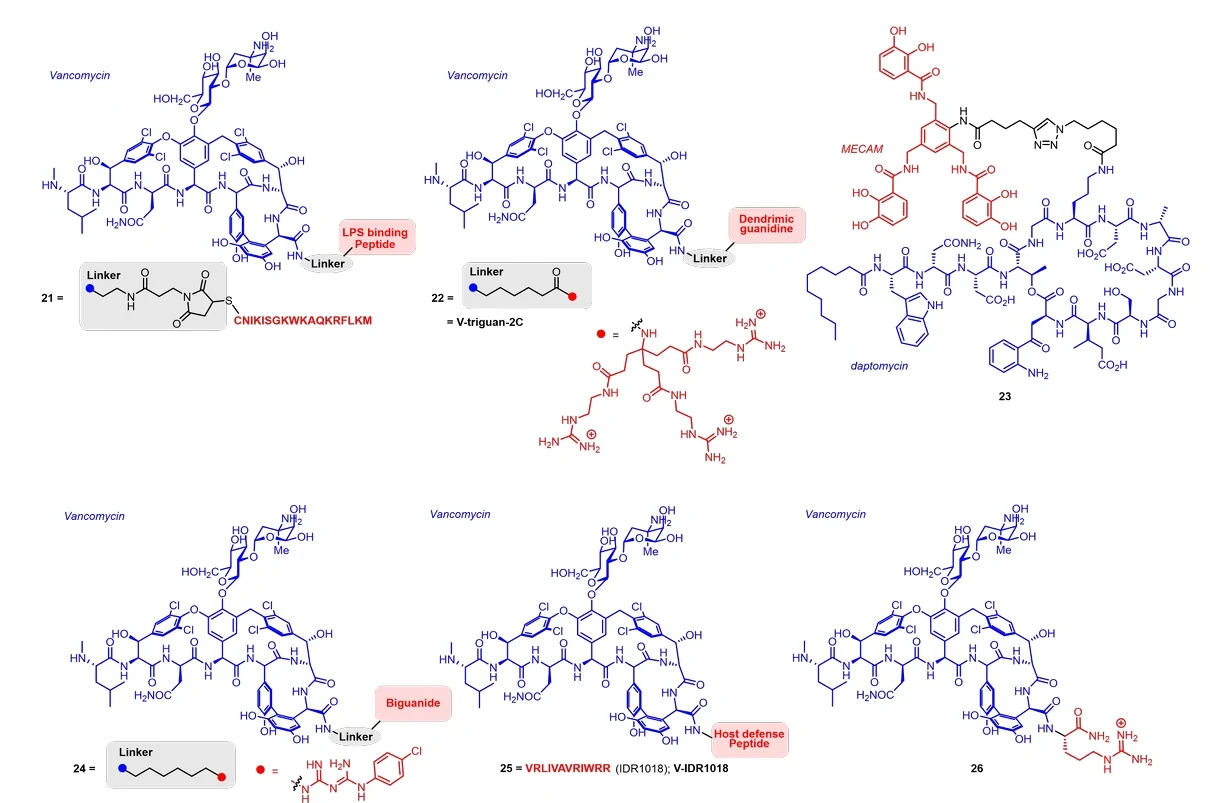
Recent examples of vancomycin-based antibiotic hybrids.

**Fig. 4.**
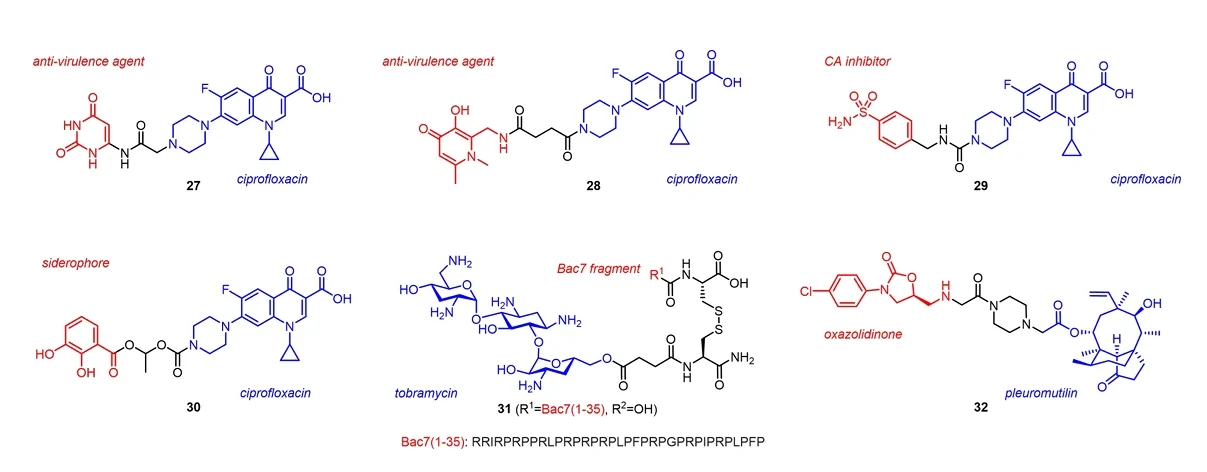
Recent examples of non-β-lactam-based antibiotic hybrids.

Beyond the β-lactam class, hybrid strategies have been extensively applied to other major antibiotic families, particularly glycopeptides, fluoroquinolones, and lipopeptides. These recent advancements demonstrate the versatility of the hybrid strategy in broadening the antimicrobial spectrum, combating resistance mechanisms, and eradicating persistent biofilms (Figs. 3 and 4, Table 2).

A significant breakthrough involves expanding the spectrum of Gram-positive-specific antibiotics, such as vancomycin and daptomycin, to target Gram-negative bacteria by overcoming the OM barrier. Shi et al. (2021) developed vancomycin-LPS binding peptide conjugates, exemplified by Compound 21. By incorporating a cationic peptide that specifically interacts with lipopolysaccharides (LPS), this hybrid successfully permeabilized the OM, achieving potent activity against *E. coli* and *A. baumannii*. Similarly, Chosy et al. (2024) employed a dendrimeric approach, synthesizing vancomycin-poly-guanidino dendrimer conjugates (Compound 22). The multivalent display of guanidinium groups facilitated efficient OM penetration, resulting in significant bactericidal activity against Gram-negative pathogens while maintaining a favorable safety profile. This spectrum-expanding strategy was also applied to lipopeptides; Pinkert et al. (2021) conjugated daptomycin with a MECAM siderophore to create Compound 23. This modification successfully sensitized Gram-negative *A. baumannii* to daptomycin, a drug typically restricted to Gram-positive infections.

In addition to spectrum expansion, hybrids have been strategically designed to combat resistance mechanisms and biofilm formation. Addressing vancomycin-resistant enterococci (VRE), Rahn et al. (2024) introduced Compound 24, a vancomycin-biguanide conjugate. The hydrophobic biguanide tail enhanced membrane interaction, not only restoring potency against VRE but also demonstrating broad-spectrum anti-biofilm activity. Furthermore, Etayash et al. (2021) conjugated vancomycin with the innate defense regulator peptide IDR1018 (Compound 25), creating a multimodal hybrid that eradicated MRSA biofilms and modulated the host immune response. Expanding the target range to mycobacteria, Brčić et al. (2023) synthesized a vancomycin-arginine conjugate (Compound 26), which showed enhanced efficacy against *Mycobacterium abscessus* by promoting specific interactions with mycobacterial peptidoglycan.

For fluoroquinolone-based hybrids, Samir et al. (2022) developed ciprofloxacin-uracil conjugates (Compound 27), designed to inhibit both DNA gyrase and topoisomerase IV. This dual inhibition mechanism proved effective against resistant MRSA strains where ciprofloxacin alone had failed. Targeting bacterial virulence, Wang et al. (2023) conjugated ciprofloxacin with a hydroxypyridinone-based anti-virulence agent (Compound 28). While maintaining antibacterial efficacy, this compound significantly inhibited biofilm formation and virulence factor production in *P. aeruginosa*, offering a dual-pronged approach to infection control. Similarly, Marinacci et al. (2024) combined ciprofloxacin with a carbonic anhydrase inhibitor (Compound 29), which demonstrated potent activity against *P. aeruginosa* biofilms. Addressing drug delivery challenges, Loupias et al. (2023) explored siderophore-ciprofloxacin conjugates, identifying that the incorporation of a cleavable linker (Compound 30) is essential for intracellular drug release and subsequent antibacterial activity against *B. pseudomallei*.

Finally, recent studies have emphasized the critical roles of transporter specificity and synergistic combinations. Gambato et al. (2023) developed tobramycin-peptide conjugates, exemplified by Compound 31, which utilize the SbmA transporter for bacterial entry. Notably, the activity of these hybrids was significantly reduced in *sbmA* mutant strains, confirming a transporter-dependent uptake mechanism distinctive from free tobramycin. Additionally, Xia et al. (2023) reported a pleuromutilin-oxazolidinone hybrid (Compound 32) that exhibited superior potency against MDR *S. aureus* compared to either parent drug, highlighting the synergistic potential of combining distinct protein synthesis inhibitors.

## Antibiotic Hybrids in Clinical Trials

**Fig. 5.**
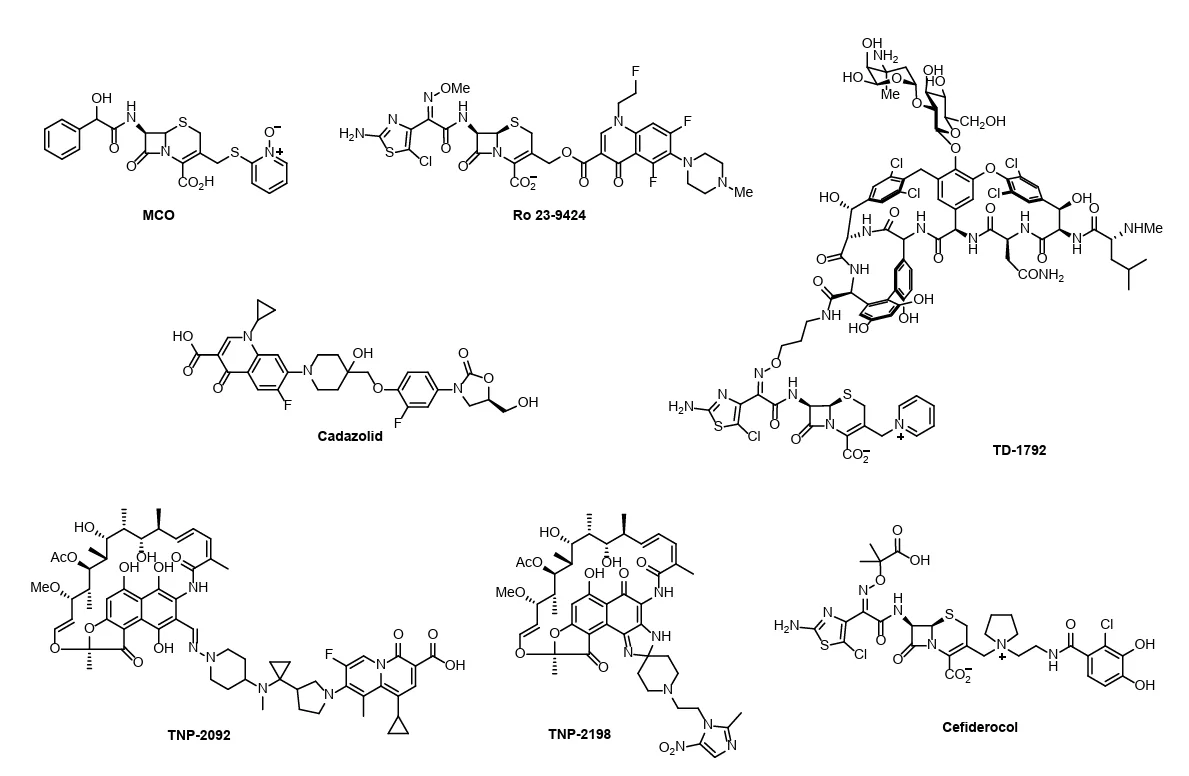
Chemical structures of antibiotic hybrids in clinical development.

As summarized in Table 3, several antibiotic hybrids have progressed to clinical evaluation (Fig. 5). This section reviews representative candidates in chronological order of their development, highlighting their design rationale and clinical outcomes.

### MCO (1976)

Early efforts in hybrid antibiotic development were inspired by the mechanism of cephalosporins, which expel a leaving group at the 3-position upon β-lactamase-mediated hydrolysis (Hamilton-Miller et al., 1970; O'Callaghan et al., 1972). Leveraging this mechanism, O'Callaghan et al. (1976) designed MCO by linking the sulfur atom of 2-mercaptopyridine-N-oxide (omadine) to the 3-position of a cephalosporin core. While the released omadine exhibited potent antimicrobial activity, the compound’s development was halted due to the systemic toxicity associated with the free omadine moiety. Nevertheless, MCO served as a pioneering proof-of-concept for cephalosporin-based dual-action prodrugs.

### Ro 23-9424 (1990)

Developed by Roche, Ro 23-9424 is a cephalosporin-fluoroquinolone hybrid featuring an ester linkage between the C-3 position of desacetylcefotaxime and the carboxylic acid of fleroxacin (Albrecht et al., 1990; Bryskier, 1997). The hybrid demonstrated broad-spectrum activity against both Gram-positive and Gram-negative bacteria, including strains resistant to cephalosporins or fluoroquinolones (Jones et al., 1989). Pharmacokinetic studies confirmed that the molecule is primarily excreted intact, demonstrating that the ester linkage retains sufficient stability in vivo, although partial hydrolysis to its active components also occurs (Beskid et al., 1990; Christenson et al., 1990). However, clinical development was discontinued after Phase I, likely because the hybrid failed to demonstrate a significant therapeutic advantage over the co-administration of its individual components (Domalaon et al., 2018).

### TD-1792 (2008)

TD-1792, developed by Theravance Therapeutics, is a multivalent heterodimer consisting of a vancomycin core covalently linked to a cephalosporin oxime moiety (Long et al., 2008). This design aimed to combine the cell wall synthesis inhibition of both pharmacophores. TD-1792 exhibited potent bactericidal activity against Gram-positive pathogens, including MRSA and vancomycin-intermediate *S. aureus* (VISA) (Blais et al., 2012; Leuthner et al., 2010), with superior in vivo efficacy compared to vancomycin in a neutropenic murine thigh infection model (Hegde et al., 2012). In Phase II clinical trials, TD-1792 achieved clinical cure rates comparable to vancomycin (91.7% vs. 90.7%) for complicated skin and skin structure infections (Stryjewski et al., 2012). The drug is currently in Phase III trials in Russia and Georgia.

### Cadazolid (2014)

Cadazolid is a quinolonyl-oxazolidinone hybrid developed for the treatment of CDI (Rueedi et al., 2024). It was designed to inhibit protein synthesis via the oxazolidinone pharmacophore while minimizing the risk of resistance through the fluoroquinolone moiety. Cadazolid demonstrated potent activity against *C. difficile* strains, including those resistant to linezolid and moxifloxacin (Locher et al., 2014a, 2014b). Notably, cadazolid exhibits limited systemic exposure and is excreted largely unchanged in feces, ensuring high local concentrations in the colon essential for treating CDI (Baldoni et al., 2014). Although Phase II trials showed promising clinical response rates comparable to vancomycin, Phase III trials failed to demonstrate non-inferiority to vancomycin, leading to the discontinuation of its development (Gerding et al., 2019; Louie et al., 2015).

### TNP-2092 (2019)

TNP-2092 (formerly CBR-2092) is a rifamycin-quinolizinone conjugate developed by TenNor Therapeutics, currently in Phase III trials for prosthetic joint infections (PJIs) (Ma and Lynch, 2016). While rifamycin is highly effective against biofilm-embedded bacteria, its utility is often limited by the rapid emergence of resistance due to point mutations in RNA polymerase. TNP-2092 addresses this limitation by tethering a quinolizinone pharmacophore, which targets DNA gyrase and Topoisomerase IV. This dual mechanism of action—inhibiting RNA polymerase, DNA gyrase, and Topoisomerase IV—results in a significantly lower frequency of resistance (≈ 10^-12^) compared to rifamycin alone (O’Neill et al., 2001). The compound demonstrates a favorable pharmacokinetic profile with significant tissue accumulation, supporting its evaluation for treating chronic prosthetic joint infections involving biofilms (Ma and Lynch, 2016).

### Cefiderocol (2019)

Cefiderocol is a first-in-class siderophore-cephalosporin conjugate approved for the treatment of cUTIs and HAP caused by multidrug-resistant Gram-negative bacteria (Sato and Yamawaki, 2019). The molecule features a catechol-based siderophore moiety linked to the cephalosporin core, allowing it to actively cross the OM via bacterial iron transport channels—a strategy known as the "Trojan horse" approach. This active transport mechanism improves periplasmic entry and helps overcome permeability barriers, including porin loss and efflux. Additionally, the structural features of cefiderocol provide marked stability against a broad range of β-lactamases.

Its pharmacokinetics/pharmacodynamics (PK/PD) index is best described by the percentage of time that free drug concentrations exceed the MIC (%fT > MIC), consistent with other β-lactam antibiotics (Bilal et al., 2021). Cefiderocol has demonstrated non-inferiority to standard therapies in Phase III trials (e.g., the APEKS-NP trial) and remains a critical option for treating carbapenem-resistant pathogens (Tamma et al., 2024).

### TNP-2198 (2022)

Building on the dual-targeting strategy of TNP-2092, TNP-2198 (rifasutenizol) is a rifamycin-nitroimidazole hybrid designed for targeting microaerophilic and anaerobic bacteria such as *H. pylori* and *C. difficile* (Ma et al., 2022). The rifamycin core inhibits RNA polymerase, while the nitroimidazole moiety causes DNA damage after reductive activation, effectively suppressing resistance through synergistic action. While Phase I and II evaluations established a favorable safety profile and demonstrated high eradication rates in preliminary trials (Li et al., 2024), the recent Phase III EVEREST-HP trial has definitively confirmed the clinical efficacy of TNP-2198-based triple therapy, demonstrating non-inferiority to standard treatments and highlighting its potential as a next-generation therapy for resistant infections (Song et al., 2025).

## Future Outlooks

Antibiotic hybrids represent a promising solution to overcoming the limitations of the current antibiotic therapies. By combining dual pharmacophores, hybrids can effectively evade AMR by counteracting resistance mechanisms that target single pharmacophores. Despite significant progress, further scientific innovation is required to strengthen the antibiotic pipeline.

One limitation of antibiotic hybrids is their large molecular weight, which restricts their effectiveness to Gram-positive bacteria, except for siderophore-conjugate hybrids. To fully exploit dual-targeting strategies against Gram-negative bacteria, this challenge must be overcome. Siderophore conjugation can be an effective strategy to overcome this limitation. For example, siderophore conjugation to a cephalosporin-oxazolidinone hybrid improved antimicrobial activity against Gram-negative bacteria, whereas the unconjugated form showed no activity (Liu et al., 2018).

Currently, hybrid antibiotic strategies primarily rely on well-established classes of antibiotics. Integrating these with novel targets emerging from recent antibiotic pipelines offers a unique opportunity to address AMR threats more comprehensively. Furthermore, there is considerable potential for advancing linker technology. Lessons from fields such as PROTACs and antibody-drug conjugates reveal that precise optimization of linker components can significantly enhance the physicochemical properties and efficacy of drugs.

Despite its current limitations, the hybrid strategy represents a promising approach to addressing the evolving threat of AMR. The development of entirely new antibiotics with novel modes of action remains the ultimate goal; however, the long timeline required for such breakthroughs underscores the significance of antibiotic hybrids as a practical and pivotal solution. These compounds can help bridge the discovery gap, providing a steady supply of antibiotic candidates to sustain the pipeline until new drugs are approved. To ensure the successful development of hybrid antibiotics and their progression to clinical use, focused research efforts are needed to refine linker designs, deepen our understanding of their molecular mechanisms, and optimize their PK and PD properties.

## Figures and Tables

**Table 1.** Representative β-lactam-based antibiotic hybrids and their comparative efficacy

Cpd.	Hybrid design	Target	Efficacy	Reference
1	Amoxicillin + CA inhibitor	*N. gonorrhoeae* (CDC-178)	MIC, 1 (1) vs 2 (amoxicillin) μg/ml	Bonardi et al. (2024)
2	Carbapenem + MBL Inhibitor	MBL Enzymes (NDM-1, IMP-1)	High Enzyme Inhibition (IC_50_ nM range), Inactive in cells	Gao et al. (2024)
3	Cephalosporin + 8-TQ	*K. pneumoniae (MBL-producer)*	Restored efficacy of Meropenem via enzyme-triggered release of 8-TQ.	van Haren et al. (2021)
6	Meropenem + Enterobactin	*E. coli *(CFT073)	MIC, 0.01 (6) vs 0.1 (Meropenem) μM	Sargun et al. (2021)
8	Cefaclor + Fimsbactin B	*A. baumannii *(ATCC 17978)	MIC, 0.5 (8) vs 64 (Cefaclor) μg/ml	Kim and Kim (2021)
9	Ampicillin + TRENCAM	*S*. Typhimurium	MIC, 0.01 (9) vs 10 (Ampicillin) μM	Motz et al. (2024)
10	Ampicillin + MECAM	*E. coli*	MIC, 1.5 (10) vs 46 (Ampicillin) μM	Pinkert et al. (2021)
11	Cephalosporin + Catechol	*A. baumannii* (MDR Isolate)	MIC, 0.25 (11) vs ≥ 64 (Meropenem) μg/ml	Liu et al. (2024)
20	Cephalosporin + POA	*M. tuberculosis* (H37Rv Δ*pncA*)	MIC, 100 (20) vs > 800 (PZA) vs 100 (POA) μg/ml	Cole et al. (2022)

**Table 2.** Representative non-β-lactam-based antibiotic hybrids and their comparative efficacy

Cpd.	Hybrid design	Target	Efficacy	Reference
21	Vancomycin + LPS-binding peptide	*E. coli *(AB1157)	MIC, 8 (21) vs > 88 (Vancomycin) μM	Shi et al. (2021)
22	Vancomycin + Dendrimeric guanidine	*E. coli *(UTI89)	MIC, 8 (22) vs 128 (Vancomycin) µM	Chosy et al. (2024)
23	Daptomycin + MECAM	*A. baumannii*	MIC, 4.4 (23) vs > 39 (Daptomycin) µM	Pinkert et al. (2021)
24	Vancomycin + Biguanide	*E. faecium *(ATCC 51559)	MIC, 4 (24) vs 512 (Vancomycin) µM	Rahn et al. (2024)
25	Vancomycin + IDR1018 peptide	MRSA biofilms	Minimal biofilm eradication conc. 16 (25) vs > 128 (Vancomycin) µg/ml	Etayash et al. (2021)
26	Vancomycin + Arginine	*M. abscessus *(ATCC 19977)	MIC, 16 (26) vs 64 (Vancomycin) µM	Brčić et al. (2023)
27	Ciprofloxacin + Uracil	MRSA (AUMC261)	MIC, 0.031 (27) vs 0.57 (Ciprofloxacin) µM	Samir et al. (2022)
28	Ciprofloxacin + Anti-virulence agent	*P. aeruginosa* Biofilms	Biofilm reduction at 1/4 MIC, 78.3% (28) vs 12.6% (Ciprofloxacin)	Wang et al. (2023)
30	Ciprofloxacin + Siderophore	*B. pseudomallei *(BPS2020IRBA003)	MIC, 2 (30) vs 16 (Ciprofloxacin) µg/ml	Loupias et al. (2023)
31	Tobramycin + Bac7 peptide fragment	*E. coli *(BW25113)	MIC, 1 (31) vs 4 (Tobramycin) µM	Gambato et al. (2023)
32	Pleuromutilin + Oxazolidinone	MRSA (ATCC33591)	MIC, 0.063 (32) vs > 0.5 (Pleuromutilin) μg/ml	Xia et al. (2023)

**Table 3.** Overview of antibiotic hybrids in clinical development

Name (Year)	Hybrid design	Target	Current status	Features
MCO (1976)	Cephalosporin + Omadine	G(+) & G(-)	Discontinued	Demonstrated release of active omadine upon β-lactamase hydrolysis, but halted due to systemic toxicity
Ro 23-9424 (1990)	Desacetylcefotaxime (C-3) + Fleroxacin	G(+) & G(-)	Discontinued	Broad-spectrum ester-linked prodrug excreted intact. Discontinued due to lack of therapeutic advantage over co-administration.
TD-1792 (2008)	Vancomycin + Cephalosporin	G(+) (MRSA)	Phase 3	Dual-targeting mechanism inhibiting both Lipid II and PBPs. Showed non-inferiority to vancomycin with high bactericidal activity in Phase II.
Cadazolid (2014)	Oxazolidinone + Fluoroquinolone	*Clostridioides difficile* infection (CDI)	Discontinued	Low systemic absorption ideal for CDI. Discontinued after failing to demonstrate non-inferiority in Phase III
TNP-2092 (2016)	Rifamycin + Quinolizinone	*H. pylori*, Biofilms	Phase 3	Dual inhibition (RNA polymerase + Gyrase/Topo IV) minimizes resistance frequency. Effective against persistent biofilms.
Cefiderocol (2019)	Siderophore + Cephalosporin	MDR G(-)	Approved	Active transport via iron channels overcomes OM barrier. Stable against carbapenemases.
TNP-2198 (2022)	Rifamycin + Nitroimidazole	Anaerobes (*H. pylori*)	Phase 3	Multi-targeting of microaerophiles. Recent trials confirmed high eradication rates in *H. pylori* triple therapy.
